# Efficiency of Our Health Laws

**Published:** 1900-10

**Authors:** 


### Efficiency of Our Health Laws.

H. W. Chapman, M. D., in an article read before a special meeting of the Morgan County Medical Society, says:

"Under existing national health laws, the local authorities must demonstrate their inefficiency, before the national authority can intervene, which defeats the very purpose for which such laws were enacted. The United States Marine Hospital Service constitutes a very efficient national health board if given proper authority.

"The law establishing our State board of health briefly clothes it with all the power of the State to enforce its regulations, and the mandates of this board is the law from which there is no appeal.

Local boards of health are appointed by the local authorities, and are clothed with all the power of the appointing officers to enforce their edicts. These boards are usually appointed from members of the city council, and generally have but little knowledge of sanitation. Things offensive to sight and smell are not always a menace to health excepting in the minds of these boards. Things that offend neither of these senses may be of the greatest danger, and are uniformly passed over.

"The pollution of streams with sewage should be absolutely prohibited. The excavations of out-houses, instead of being covered with earth when full, and the house moved over a new excavation, should be cemented water tight and cleaned out by the city at public expense every spring, thus keeping the ground free from excrement and the water passing through it to the wells pure. This material can be composted and form a valuable fertilizer."—*Medical Progress.*

